# A Comparative Study of Serum Exosome Isolation Using Differential Ultracentrifugation and Three Commercial Reagents

**DOI:** 10.1371/journal.pone.0170628

**Published:** 2017-01-23

**Authors:** Inas Helwa, Jingwen Cai, Michelle D. Drewry, Arthur Zimmerman, Michael B. Dinkins, Mariam Lotfy Khaled, Mutsa Seremwe, W. Michael Dismuke, Erhard Bieberich, W. Daniel Stamer, Mark W. Hamrick, Yutao Liu

**Affiliations:** 1 Department of Cellular Biology and Anatomy, Augusta University, Augusta, Georgia, United States of America; 2 Department of Neuroscience and Regenerative Medicine, Augusta University, Augusta, Georgia, United States of America; 3 Department of Ophthalmology, Duke University, Durham, North Carolina, United States of America; 4 Department of Biomedical Engineering, Duke University, Durham, North Carolina, United States of America; 5 James & Jean Culver Vision Discovery Institute, Augusta University, Augusta, Georgia, United States of America; 6 Center for Biotechnology and Genomic Medicine, Augusta University, Augusta, Georgia, United States of America; Universita degli Studi di Torino, ITALY

## Abstract

Exosomes play a role in cell-to-cell signaling and serve as possible biomarkers. Isolating exosomes with reliable quality and substantial concentration is a major challenge. Our purpose is to compare the exosomes extracted by three different exosome isolation kits (miRCURY, ExoQuick, and Invitrogen Total Exosome Isolation Reagent) and differential ultracentrifugation (UC) using six different volumes of a non-cancerous human serum (5 ml, 1 ml, 500 μl, 250 μl, 100 μl, and 50 μl) and three different volumes (1 ml, 500 μl and 100 μl) of six individual commercial serum samples collected from human donors. The smaller starting volumes (100 μl and 50 μl) are used to mimic conditions of limited availability of heterogeneous biological samples. The isolated exosomes were characterized based upon size, quantity, zeta potential, CD63 and CD9 protein expression, and exosomal RNA (exRNA) quality and quantity using several complementary methods: nanoparticle tracking analysis (NTA) with ZetaView, western blot, transmission electron microscopy (TEM), the Agilent Bioanalyzer system, and droplet digital PCR (ddPCR). Our NTA results showed that all isolation techniques produced exosomes within the expected size range (40–150 nm). The three kits, though, produced a significantly higher yield (80–300 fold) of exosomes as compared to UC for all serum volumes, except 5 mL. We also found that exosomes isolated by the different techniques and serum volumes had similar zeta potentials to previous studies. Western blot analysis and TEM immunogold labelling confirmed the expression of two common exosomal protein markers, CD63 and CD9, in samples isolated by all techniques. All exosome isolations yielded high quality exRNA, containing mostly small RNA with a peak between 25 and 200 nucleotides in size. ddPCR results indicated that exosomes isolated from similar serum volumes but different isolation techniques rendered similar concentrations of two selected exRNA: hsa-miR-16 and hsa-miR-451. In summary, the three commercial exosome isolation kits are viable alternatives to UC, even when limited amounts of biological samples are available.

## Introduction

Extracellular vesicles are spherical particles with phospholipid bilayers released by various cell types *in vivo* into body fluids such as serum, urine, cerebrospinal fluid, breast milk, aqueous humor, and amniotic fluid [1–7], as well as *in vitro* by cultured cells [8]. It is becoming increasingly obvious that these vesicles are pivotal mediators of cell-cell communication in multicellular organisms, having pleiotropic cellular and biological functions [9–14]. Hence, they are now regarded as multifunctional signaling complexes and major contributors to disease pathways such as tumor progression and metastasis [15]. Generally, extracellular vesicles are classified according to their cellular origin and biogenesis into microvesicles, exosomes, and apoptotic bodies [16]. Exosomes range in size from 40–150 nm, and they are derived from the endosomal compartment within the cell [17]. Exosomal content includes genomic DNA, RNA, proteins, and lipids [10, 13, 15, 18, 19]. Over the past decade, exosomes have gained specific interest as microRNA (miRNA) carriers, disease biomarkers, and potential therapeutic targets [17, 20, 21]. Despite their importance, exosome isolation and characterization are still considered major scientific challenges [22, 23], and identifying the optimal technique to isolate exosomes is essential for further biomarker discoveries.

The traditional differential ultracentrifugation (UC) has been widely adapted as a reliable technique for isolating exosomes from biological fluids [24]. Recently, a number of commercial kits have been launched to isolate and study exosomes for various purposes [25–27]. Compared to UC, these kits are less time consuming, less technique sensitive, more compatible with limited volumes of biological samples, and do not require special equipment. Prior to downstream proteomic and genomic analyses using exosomes isolated by these methods, though, comprehensive characterization using parameters such as size, yield, zeta potential, and exosomal RNA (exRNA) quality and quantity is necessary [28, 29].

Nanoparticle tracking analysis (NTA) has been used since 2006 as a credible method to measure the size and concentration of nanoparticles, including exosomes [30]. The ZetaView (Particle Metrix, Meerbusch, Germany) is a newly launched instrument capable of characterizing nanoparticles within about 10 to 2000 nm, using a laser scattering video microscope to track the movement of individual nanoparticles under Brownian motion [30–33]. Besides measuring size and concentration, the ZetaView can also be used to measure the zeta potential, which is defined as the electro-kinetic potential difference between the fixed boundary layer of a charged particle and the migrating ions in the bulk solution and is typically measured in mV [34, 35]. Being used as an indicator of stability, the higher the magnitude of the zeta potential, the higher the repulsion between the particles in solution, suggesting a reduced likelihood of agglomeration or sedimentation in the solution [32–36].

Several studies have attempted to compare the efficiency, reproducibility, and effect on downstream analyses of various exosomes isolation techniques [37–46]. Many of these reports inadequately characterized the exosomes either in terms of physical properties (size, concentration, and zeta potential) or in terms of the exRNA quality and quantity [37–42]. For example, Rekker et al. compared UC and ExoQuick using a single volume of serum samples (1 ml) in terms of miRNA expression; however, they overlooked the quality and absolute quantity of the extracted exRNA [39]. Alvarez et al. also compared UC to ExoQuick using two large volumes of urine samples (25 and 10 ml) without including any NTA data to confirm the physical properties of the isolated particles [37]. Instead, they relied exclusively on comparing the protein content of the isolated extracts, including levels of CD9, TSG101, and ALIX. These protein markers cannot be used for absolute quantification of exosomes [37, 40, 47]. In addition, the quantitative real-time PCR (qRT-PCR) data published by this group are hard to interpret due to lack of a reliable housekeeping gene that can be used to assess exRNA [37].

Therefore, the main goal of our study is to compare different isolation techniques by characterizing the exosomes using more comprehensive terms: size and morphology with NTA and transmission electron microscopy (TEM) imaging, yield with NTA, protein expression with TEM imaging and western blot analysis, zeta potential with NTA, exRNA quality with the Agilent Bioanalyzer instrument, and absolute quantity of selected miRNAs with droplet digital PCR (ddPCR).

## Materials and Methods

### Materials

Pooled human serum (Valley Biomedical, Winchester, VA, catalog #HS1004P) and six commercially available individual donor samples (BioreclamationIVT, NY, Item #HMSRM) were used for exosome isolation. We used three exosome isolation kits for this study: miRCURY^TM^ exosome isolation kit-serum and plasma (miRCURY) (Exiqon, Woburn, MA), ExoQuick^TM^ Serum Exosome Precipitation Solution (ExoQuick) (System Biosciences, Mountain view, CA), and Total Exosome Isolation Reagent for serum (TEIR) (Life Technologies, Carlsbad, CA). To isolate exRNA, miRCURY RNA Isolation Kit–Cell & Plant (Exiqon, Woburn, MA) was used. RIPA lysis and extraction buffer (GBiosciences, St. Louis, MO), 2xLaemelli buffer (Bio-Rad, Hercules, California), and 100x halt protease inhibitor EDTA-free cocktail (Thermo Scientific, Grand Island, NY) were used to prepare protein samples for western blot analysis. Rabbit monoclonal anti-CD63 antibody was from Abcam (Cambridge, MA), and rabbit polyclonal anti-CD9 antibody was purchased from Santa Cruz (Dallas, Texas). IRDye 800CW-conjugated goat anti-rabbit secondary antibody was from LI-COR (Lincoln, Nebraska).

### Exosome Isolation

Total exosomes were extracted from six different volumes of commercially available pooled human serum (5ml, 1ml, 500 μl, 250 μl, 100 μl, and 50 μl) and three different volumes of six individual donors’ serum samples (1 ml, 500 μl, and 100 μl) using TEIR, ExoQuick, miRCURY, and UC. Each volume of the pooled serum was isolated three separate times per each technique, and data were presented as the mean of these three independent experimental replicates, whereas each volume of the individual serum samples was isolated and measured once. The exosome isolation kits were used according to the manufacturer’s instructions. In brief, an initial spin was performed at 10,000xg (room temperature) for 10 minutes for each sample to remove cells and debris, then the corresponding amounts of reagents were added proportional to the starting sample volume, according to the manufacturer’s instructions. Mixtures were vortexed and incubated at 4°C for up to an hour and then centrifuged at room temperature to precipitate exosome pellets. Regarding the centrifugation parameters, samples extracted by TEIR (catalog# 4478360) were centrifuged at 10,000g for 30 minutes followed by pellet resuspension in 1XPBS. As for ExoQuick (Catalog# EXOQ5A-1) and miRCURY (catalog# 300101), centrifugation was done for 30 minutes at 1,500xg followed by pellet resuspension in nuclease free water and the manufacturer-supplied resuspension buffer, respectively. The resuspension volume for exosome pellets was 500 μl for 5 ml and 100 μl for 1 ml starting volumes. To ensure enough concentration of exosomes for further analysis, all other starting volumes were resuspended in 50 μl buffer or nuclease free water. All exosomes were stored at -80°C immediately after isolation until further analysis. For NTA, all samples were diluted using 1XPBS, and resuspension volumes and dilution factors were used to calculate the total number of isolated exosomes, shown in **Table 1**. For TEM, all samples were freshly isolated and diluted in nuclease-free water for better image quality.

**Table 1:** Nanoparticle tracking analysis of exosomes isolated from the commercial pooled, non-cancerous human serum using various techniques and serum volumes.

Serum Volume	Isolation Technique	Mode/Diameter* (nm) (mean ± SEM)	Total Number of particles* (mean ± SEM)
5 mL	miRCURY	122 ± 10	7.3x10^11^ ± 3.0x10^11^
	ExoQuick	111 ± 5	7.4x10^11^ ± 3.5x10^11^
	TEIR	105 ± 5	9.9x10^11^ ± 4.2x10^11^
	UC	134 ± 3	2.4x10^9^ ± 1.1x10^8^
1 mL	miRCURY	106 ± 3	1.5x10^11^ ± 2.6x10^10^
	ExoQuick	109 ± 4	1.8x10^11^ ± 3.1x10^10^
	TEIR	112 ± 4	1.4x10^11^ ± 5.9x10^10^
	UC	133 ± 11	1.1x10^9^ ± 2.0x10^8^
500 μL	miRCURY	116 ± 5	4.5x10^10^ ± 8.9x10^9^
	ExoQuick	117 ± 8	6.6x10^10^ ± 1.2x10^10^
	TEIR	107 ± 3	1.0x10^11^ ± 2.6x10^10^
	UC	126 ± 2	4.6x10^8^ ± 8.5x10^7^
250 μL	miRCURY	118 ± 5	3.0x10^10^ ± 4.6x10^9^
	ExoQuick	119 ± 1	4.2x10^10^ ± 1.5x10^10^
	TEIR	122 ± 3	2.4x10^10^ ± 5.8x10^9^
	UC	129 ± 7	2.9x10^8^ ± 7.5x10^7^
100 μL	miRCURY	119 ± 5	1.1x10^10^ ± 1.6x10^9^
	ExoQuick	126 ± 2	4.1x10^10^ ± 1.5x10^10^
	TEIR	125 ± 3	1.8x10^10^ ± 5.6x10^9^
	UC	126 ± 4	1.2x10^8^ ± 3.5x10^7^
50 μL	miRCURY	117 ± 6	5.5x10^9^ ± 8.5x10^8^
	ExoQuick	118 ± 2	6.5x10^9^ ± 1.8x10^9^
	TEIR	119 ± 4	6.6x10^9^ ± 1.5x10^9^
	UC	123 ± 4	7.0x10^8^ ± 3.2x10^7^
Average^§^	miRCURY	116 ± 3	
	ExoQuick	117 ± 3	
	TEIR	115 ± 4	
	UC	129 ± 2	

* Data included in this table represent the mean readings of three experimental replicates that were extracted, diluted, and measured separately. Data intervals represent the SEM.

^§^ Refers to the average diameter of the isolated particles by each technique from all starting volumes collectively. TEIR: Total Exosome Isolation Reagent from serum (Life Technologies, Carlsbad, CA); UC, ultracentrifugation; miRCURY: miRCURY Exosome Isolation Kit-Serum and Plasma (Exiqon, Woburn, MA), ExoQuick: Exoquick Serum Exosome Precipitation Solution (System Biosciences, Mountain view, CA).

For differential ultracentrifugation, the serum samples from each starting volume were centrifuged at 20,000xg (4°C) for 30 min to remove contaminating debris. Samples were then centrifuged at 110,000xg (4°C) for 70 min using Sw55Ti rotor, and pellets were resuspended in 1XPBS. Samples were then centrifuged again at 110,000xg, after which the pellets were resuspended in 1XPBS and stored at -80°C for further analysis.

### ZetaView Nanoparticle Tracking Analysis

NTA was performed using the ZetaView PMX 110 (Particle Metrix, Meerbusch, Germany) and its corresponding software (ZetaView 8.02.28) [31]. For each sample, 2 ml of the sample, diluted in 1xPBS, was loaded into the cell, and the instrument measured each sample at 11 different positions throughout the cell, with two cycles of readings at each position. After automated analysis of all 11 positions and removal of any outlier positions, the mean, median, and mode (indicated as diameter) sizes, as well as the concentration of the sample, were calculated by the optimized machine software. For each measurement, the instrument pre-acquisition parameters were set to a temperature of 23°C, a sensitivity of 85, a frame rate of 30 frames per second (fps), a shutter speed of 100, and a laser pulse duration equal to that of shutter duration. Post-acquisition parameters were set to a minimum brightness of 25, a maximum size of 200 pixels, and a minimum size of 5 pixels. Polystyrene particles from ThermoFisher Scientific with a known average size of 100 nm were used to calibrate the instrument prior to sample readings. Automated quality control measurements including, but not limited to, cell quality check and instrument alignment and focus were also performed prior to the use of the ZetaView for sample measurements. Temperature, conductivity, electrical field, and drift measurements were recorded for further quality control. Measurement data from the ZetaView were analyzed using the corresponding software, ZetaView 8.02.28, and Microsoft Excel 2013 (Microsoft Corp., Seattle, WA, USA). As described previously, we selected the mode as the measurement for size in our analysis [13, 32, 48, 49]. The mode, also referred to as the diameter, is defined as the size of the most abundant particles. Since resuspension volume varied with technique and serum volume, resuspension volumes and dilution factors were used to convert the yield from concentration to an absolute number of particles, helping improve the accuracy and consistency of data interpretation. The number of particles per particle size curves was created using quadratic interpolation **(Table 1)**.

### ZetaView Zeta Potential Measurements

Zeta potential was measured at 23°C using the ZetaView PMX 110, as previously described [32]. Zeta potential measurements were performed using samples diluted in 0.05XPBS solution, prepared by diluting 1X PBS 20 times with DNase, RNase-free water and adjusting the solution conductivity to approximately 500 μS/cm, and the PBS pH and conductivity were monitored to ensure consistency. All of the acquisition parameters were identical to those of the size and concentration measurements. Zeta potential was measured for experimental triplicates of each starting volume of pooled human serum isolated per the different techniques, while each individual human serum was measured one time per technique and starting volume. Similar to the size and concentration measurements, the data were analyzed using the instrument software, ZetaView 8.02.28, and Microsoft Excel 2013 (Microsoft Corp., Seattle, WA, USA).

### Transmission Electron Microscopy (TEM)

For transmission electron microscopy, freshly isolated exosome suspensions were fixed in 4% paraformaldehyde for 1 hour. Exosome suspensions from different samples (approximately 5 μl) were applied to copper mesh Formvar coated carbon stabilized grids, were allowed to adsorb to the grid for 4–5 minutes and then were wicked off with filter paper. For negative staining of exosomes, 1% Aqueous Uranyl Acetate (5 μl) was applied to the grid for 30 seconds, then wicked off with Whatman filter paper. Grids were allowed to thoroughly dry before viewing.

As for immunoelectron labelling with anti-CD63 and anti-CD9, exosome samples were fixed overnight in 4% paraformaldehyde diluted in 0.1M cacodylate buffer (pH 7.4). Fixed exosome preparations (20 μl) were applied to a carbon-Formvar coated 200 mesh nickel grids, and samples were allowed to stand for 30 minutes before wiping off excess using Whatman filter paper. Grids were then floated (sample side down) onto a 20 μl drop of 1M Ammonium Chloride for 30 minutes to quench aldehyde groups from the fixation step, followed by floating on drops of blocking buffer (0.4% BSA in PBS) for 2 hours. Grids were rinsed 3 times (5 minutes each) using 1xPBS and then were allowed to incubate with either blocking buffer only (negative control) or primary antibody (CD63) diluted with blocking buffer (1:100) for 1 hour. Rinsing of the grids using deionized water (3 times for 5 minutes each) and 1xPBS followed the incubation step. Grids were then floated on drops of 1.4 nm anti-rabbit nanogold (Nanoprobes, Inc.) diluted 1:1000 in blocking buffer for 1 hour. Enhancing of grids using HQ Silver (gold enhancement reagent, Nanoprobes, Inc.) was then performed for 1 minute, followed by rinsing in deionized ice-cold water. As a final step, negative staining in 2% aqueous Uranyl Acetate was performed, and samples were wicked dry and then allowed to air dry. TEM examination was performed using JEM 1230 transmission electron microscope (JEOL USA Inc., Peabody, MA) at 110 kV and imaged with an UltraScan 4000 CCD camera & First Light Digital Camera Controller (Gatan Inc., Pleasanton, CA). TEM sample preparation and imaging was performed at the Electron Microscopy and Histology Core Laboratory at Augusta University (www.augusta.edu/mcg/cba/emhisto/).

### Western Blot Analysis

Protein samples were prepared by adding 100 μl ice-cold RIPA buffer with protease inhibitor to 100 μl extracted exosome samples suspended in the appropriate buffer. Samples were mixed by pipetting and then incubated on ice for 15 minutes; protein concentration was measured using Synergy H1 Multi-Mode Reader (BioTek, Winooski, VT, USA). 2x Laemmli buffer (100 μl) was then added, and samples were vortexed and stored at -20°C until analysis. Protein samples of 150 μg were loaded, separated on 8–10% SDS gels, and blotted on Immuno-blot PVDF membranes, followed by blocking for 1 hour and an overnight-incubation with primary antibodies at 4°C. Since the measured UC samples had such low protein content, a higher sample volume was loaded to achieve a similar protein concentration to the kit samples. This low protein content of the UC samples may be a reflection of their lower exosome yield. Membranes were then washed using Tris-buffered saline with 0.1% Tween and incubated with secondary antibody for 1 hour at room temperature. Then, membranes were washed again and visualized using infrared Odyssey machine (LI-COR Biosciences).

### RNA Isolation and Measurement

RNA isolation was performed using the miRCURY RNA Isolation Kit–Cell & Plant (catalog # 300110) (Exiqon, Woburn, MA) following the manufacturer’s instructions, as previously described [49, 50]. The selection of this kit was based on the recommendation of several publications and our own experience with different kits [51, 52]. Briefly, up to 100μl resuspended exosomes were processed with RNA isolation columns and buffers provided by the manufacturer. A final volume of 50μl RNA solution was collected from each sample using the supplied elution buffer. RNA concentration and quality were measured using Agilent 2100 Bioanalyzer (Santa Clara, CA) with the RNA 6000 Pico kit at the Integrated Genomics Core of Augusta University Cancer Center (www.augusta.edu/cancer/research/shared/genomics).

### Droplet Digital PCR (DDPCR)

The ddPCR experiments were performed using our previously published protocol [50, 53]. We selected two miRNAs, hsa-miR-16 and hsa-miR-451, which have been previously reported as being abundantly expressed in isolated serum exosomes [39, 54, 55]. Applied Biosystems (Grand Island, NY) TaqMan miRNA assays for hsa-miR-16 (catalog # 4427975, Assay ID: 000391) and hsa-miR-451 (catalog # 4427975, Assay ID: 001141) were used to examine the expression of these miRNAs in exosomes isolated from 5 ml, 1 ml, 500 μl, 250 μl, 100 μl, and 50 μl serum with the four techniques [39]. Triplicates were isolated and measured for each volume and technique. Briefly, approximately 200 pg of total RNA was reverse transcribed to cDNA using Taqman microRNA Reverse Transcriptase kit from Applied Biosystems (Grand Island, NY, USA), according to the manufacturer’s instructions. 2 μl of undiluted cDNA was used per ddPCR reaction. The reaction mix was prepared using QX200 ddPCR Supermix for probes (No dUTP) from Bio-Rad (Hercules, CA, USA), and a Bio-Rad QX200 droplet generator was used to partition each PCR reaction into up to 20,000 nano-sized droplets. The amplified PCR products were quantified using Bio-Rad QX200 droplet reader and analyzed using its associated QuantaSoft software. For quality control, ddPCR reactions with less than 10,000 droplets were excluded, and “no template controls” (ntc) were included to exclude any possible contamination.

### Statistical Analysis

Statistical analyses for particle diameter and total number of isolated particles were done using JMP Pro (SAS, Cary, NC, USA) and Microsoft Excel 2013 (Microsoft Corp., Seattle, WA, USA). Because the assumption of equal variance could not be made with our data sets, analysis of variance was done using Welch’s ANOVA [56]. Post hoc tests were conducted using two-sample unequal variance t-tests. P-values less than or equal to 0.05 were considered significant, and confidence intervals were calculated using an alpha value of 0.05. Error bars in all figures represent the standard error of the mean (SEM).

## Results

### Size and Total Number of Isolated Particles from Pooled Serum

Six different volumes of serum samples (5 ml, 1 ml, 500 μl, 250 μl, 100 μl, and 50 μl) were used for isolation, and three experimental replicates were separately isolated per each examined volume and isolation technique. The size and the total number of exosomes were comparatively analyzed using the ZetaView from Particle Matrix (**Table 1**).

**Fig 1:**
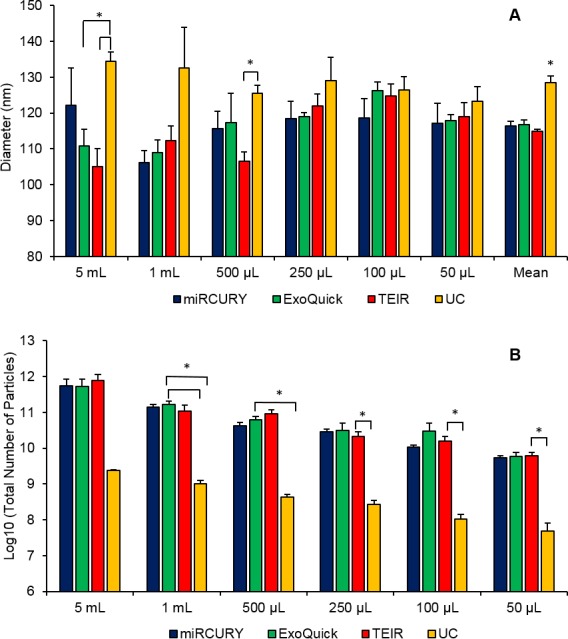
ZetaView measurements of the exosomes extracted from the six serum starting volumes of pooled human serum using the 4 different isolation techniques. NTA was done using the ZetaView instrument to measure (A) particles diameters (nm) and (B) the log10 of the total number of particles isolated using miRCURY (blue), ExoQuick (green), TEIR (red), and UC (yellow) from 5 ml, 1 ml, 500 μl, 250 μl, 100 μl, and 50 μl of human serum. The data in this graph are the mean values of three experimental replicates (±SEM), *p≤0.05.

**Fig 2:**
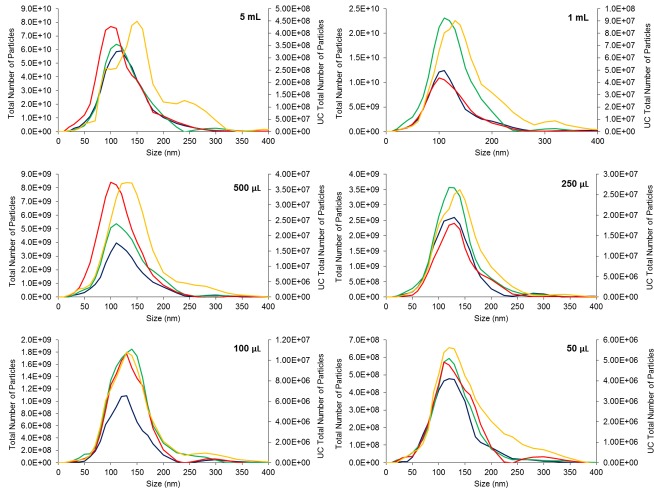
Size distribution of the exosomes isolated from pooled human serum using the different techniques and serum volumes. For each sample volume (5 ml, 1 ml, 500 μl, 250 μl, 100 μl, and 50 μl) and technique, the number of particles per a specific particle size (nm) was measured using the ZetaView for NTA. Each graph represents quadratic interpolation of the mean number of particles isolated by each technique (n = 3). Data from the commercial kits miRCURY (blue), ExoQuick (green), and TEIR (red) are graphed on the left y-axis, while the UC (yellow) data, being at a significantly lower magnitude, are mapped on the right y-axis.

According to the NTA measurements of the exosomes extracted from the pooled human serum, the isolated particles were within the expected size range for exosomes, 40–150 nm (**Fig 1A** and **Table 1**). Since no correlation was seen between starting volume and particle size, the average sizes of the most abundant particles (i.e., mode or diameter) isolated from all serum starting volumes were used to compare the different techniques. No significant differences were found between the average diameters of exosomes prepared using the different commercial kits (p > 0.05). Exosomes isolated from UC, however, had a significantly greater diameter than the three commercial kits (p = 0.003), with post hoc tests indicating a significant difference between UC and miRCURY (p = 0.003), UC and ExoQuick (p = 0.004), and UC and TEIR (p = 0.001). The increased diameter of UC isolated particles was also observed in the curves of **Fig 2**, which depict the distribution of particles across the various sizes. For most of the serum volumes, the exosome distribution curve from UC appeared shifted to the right compared to the commercial kits, suggesting larger particles sizes.

Since the number of particles isolated depended on the amount of starting material used, statistical analyses were conducted based on each starting serum volume. As shown in **Fig 1B**, while all the commercial kits generated similar yield of particles at each starting volume, UC consistently produced significantly less particles than the commercial kits for the starting volumes of 50 μL (p = 0.03), 100 μL (p = 0.04), 250 μL (p = 0.04), 500 μL (p = 0.03), and 1 mL (p = 0.03). For the 1 ml, 500 μl, 250 µl, 100 μl, and 50 μl serum starting volumes, UC isolated approximately 126-fold, 99-fold, 81-fold, 91-fold, and 79-fold less particles than the commercial kits, respectively. Despite the larger difference (~299 fold) in total particle yield between UC and the three commercial reagents with the 5 mL starting volume, this difference was not statistically significant (p > 0.05). A much larger variation was noted with the total number of particles at this starting volume for each isolation technique, which might account for the lack of significance in total number of particles. Post hoc tests revealed that for the starting volumes of 50 μL, 100 μL, and 250 μL, the statistical differences lied between miRCURY and UC, with p-values of 0.03, 0.03, and 0.03, respectively. With a serum volume of 500 μL, ExoQuick and UC only were statistically different (p = 0.04), while with 1 mL, UC was significantly different than both miRCURY (p = 0.04) and ExoQuick (p = 0.04). Consistent with these results, **Fig 2**also reflected the significantly reduced particle yield from UC compared to the commercial kits, with a much lower magnitude of particles numbers for each starting volume.

**Fig 3:**
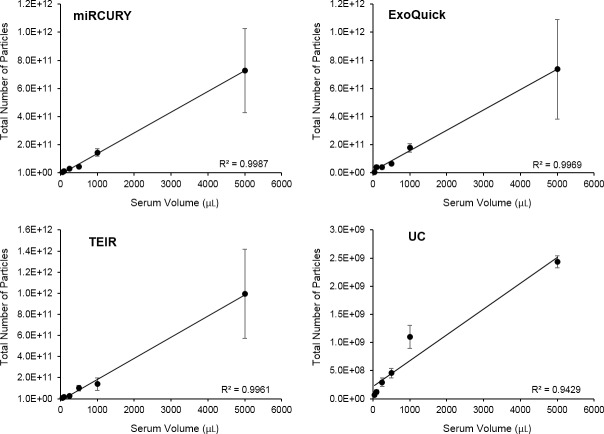
Correlation between the volume of serum and the total number of particles isolated. The relationship between the volume of serum and the total number of particles was linear for all four isolation techniques: (A) miRCURY, (B) ExoQuick, (C) TEIR, and (D) UC. The data in this graph represent the average number of isolated particles ± SEM (n = 3).

Since the relationship between the starting sample volume and the number of particles isolated is an important factor to consider in selecting the techniques, we examined this correlation in our data. As shown in **Fig 3**, the relationship between the total number of particles and the starting volume was linear for all isolation techniques, with the coefficient of determination (R^2^) indicating good linear fits for all. Large variation, though, was seen in the experimental replicates isolated using the commercial kits and the 5 ml starting volume, with SEM values ranging from 40–50% of the total number particles, as exemplified in **Fig 3**.

### Size and Total Number of Isolated Particles from Individual Serum Samples

**Fig 4:**
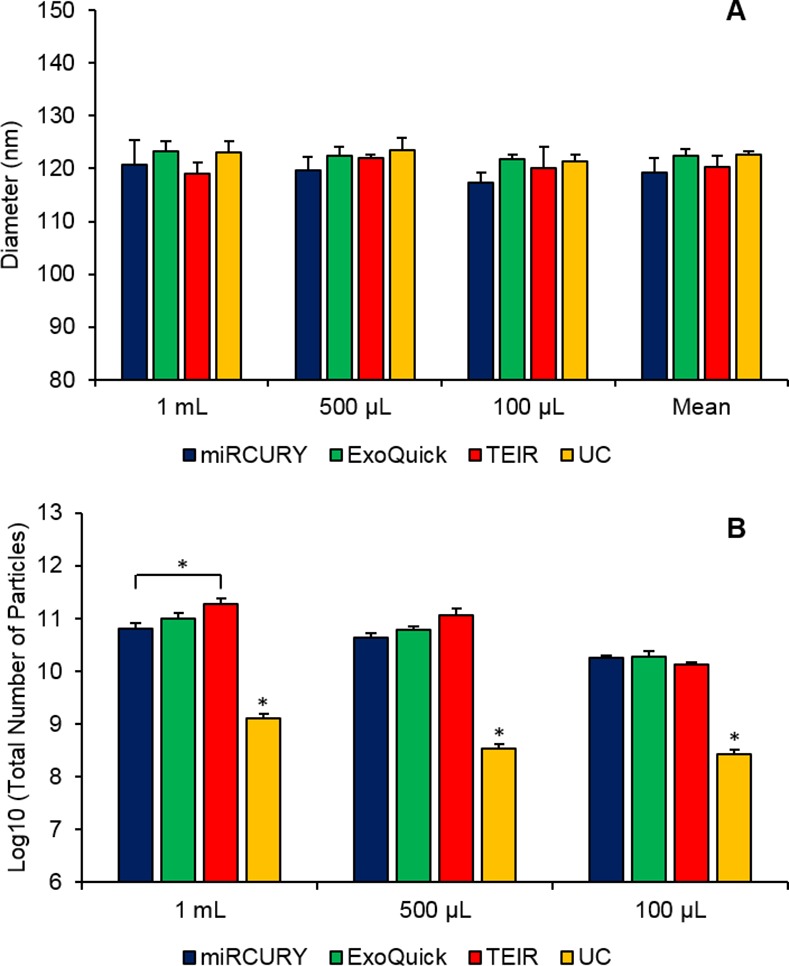
ZetaView measurements of the exosomes extracted from three different serum starting volumes of six individual human donors samples using the 4 different isolation techniques. NTA was done using the ZetaView instrument to measure (A) particles diameters (nm) and (B) the log10 of the total number of particles isolated using miRCURY (blue), ExoQuick (green), TEIR (red), and UC (yellow) from 1 ml, 500 μl, and 100 μl of human serum. The data in this graph are the mean values of the six individual human samples (±SEM), *p≤0.05.

As the previous NTA was conducted using pooled serum samples processed as three experimental replicates, we sought to confirm our results using biologically distinct samples. Accordingly, exosomes were extracted from six different human donor serum samples using the same four techniques and three serum volumes (1 ml, 500 μL, and 100 μL) that are similar to the expected quantities of human samples available in clinical studies. NTA measurements showed that the isolated particles from the individual donor serum were consistently within the expected size range for exosomes, 40–150 nm **(Fig 4)**. Unlike the pooled serum exosomes, the diameters of the individual serum particles were not statistically different (p = 0.1) between the different techniques (**Fig 4A**). Similar to the results from the pooled human serum, though, the total number of particles isolated with UC was significantly different than the commercial kits at all starting volumes (**Fig 4B**). Post-hoc tests revealed that for the 500 μL, UC was statistically different than miRCURY, ExoQuick, and TEIR with p-values of 0.01, 0.001, and 0.03, respectively, and for the the 100 μL samples, the p-values were 0.0004, 0.03, and 0.001, respectively. When comparing the number of particles isolated from 1 mL, UC remained statistically different than miRCURY (p = 0.008), ExoQuick (p = 0.006), and TEIR (p = 0.006), while TEIR and miRCURY were also significantly different (p = 0.03). Similar to the pooled serum, the kits produced more particles than UC, with at least 52-fold, 130-fold, and 44-fold more particles for the 1 mL, 500 μL, and 100 μL starting volumes, respectively.

### Morphological and Proteomic Characterization

**Fig 5:**
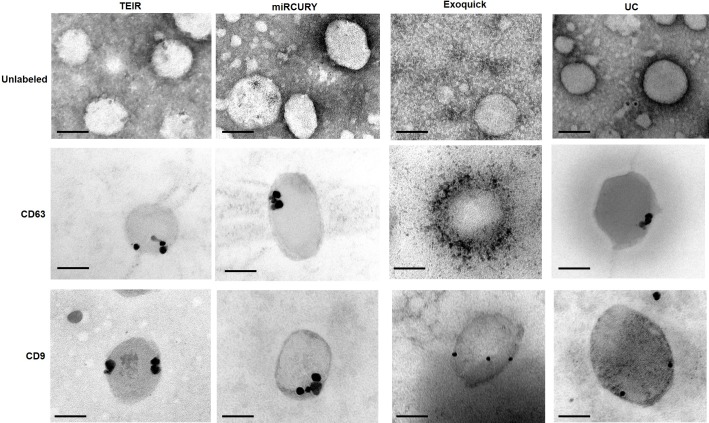
Microscopic analysis of the morphology of isolated exosomes using TEM. Upper panel shows unlabeled negative staining, middle panel shows Immunogold labeling using anti-CD63 antibody, and lower panel shows Immunogold labeling using anti-CD9 antibody. Scale bar depicts 100 nm.

After finding that the diameters of our particles are within the expected range for exosomes (40–150 nm), we further confirmed the identity of our isolates using TEM imaging, a well-accepted technique for nanoparticle validation. Negative staining (unlabeled) and immunogold labeling of the exosome samples isolated with each of the four techniques showed particles with the classical morphology of exosomes and within the anticipated size range (**Fig 5**). We then selected two proteins traditionally known to be enriched in exosomes [9, 22], the tetraspanins CD63 and CD9, and performed immunogold labelling EM (**Fig 5**) on samples isolated using the four techniques. Overall, our TEM data verified that the different techniques successfully isolated exosomes with acceptable quality in terms of size range, morphology, and exosome-specific protein enrichment (**Fig 5**).

**Fig 6:**
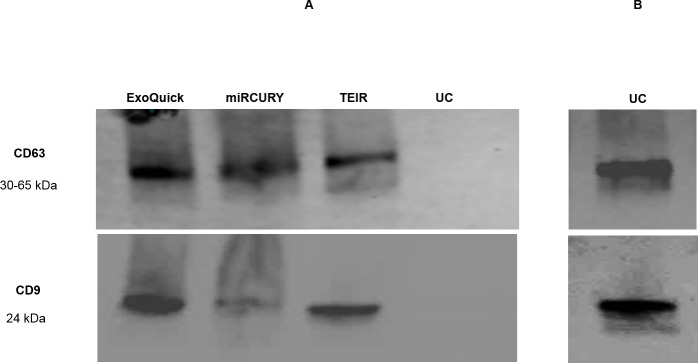
Western blot analysis of CD63 and CD9 expression in exosomes samples isolated by different techniques. The expression of the common exosomal markers CD63 and CD9 were determined using rabbit polyclonal anti-CD63 antibody and rabbit monoclonal anti-CD9 antibody. IRDye 800CW goat anti-rabbit secondary antibody and IRDye 680RD goat anti-mouse secondary antibody were used, and membranes were imaged and analyzed using LI-COR Odyssey system. (A) Expression of CD63 and CD9 using equal volumes (10 μl) of protein lysates of samples isolated by all techniques (B) Expression after loading a greater volume of protein lysate (5-fold increase; 50 μl) from the ultracentrifugation sample.

In an attempt to confirm the TEM imaging data, we probed for the tetraspanins CD63 and CD9 using western blot analysis of protein lysates prepared from the isolated exosomes. We were able to detect CD63 and CD9 expression in 10 μl of the protein lysates of the samples isolated by the commercial kits, but not in those isolated by UC (**Fig 6A**). Thus, we increased the loaded volume to 50 μl (5-fold increase) to be capable of detecting protein expression in UC samples (**Fig 6B)**. As expected, western blot analyses (**Fig 6**) demonstrated that samples from all techniques contained both CD63 and CD9 proteins.

### Zeta Potential Measurement of Isolated Exosomes

**Fig 7:**
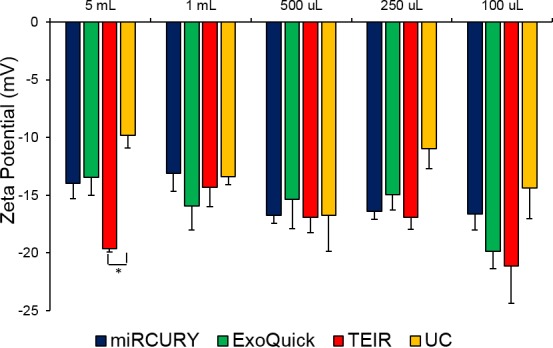
Zeta potential measurements for exosomes isolated from the different techniques and serum volumes. Using the ZetaView instrument, the zeta potential (mV) was measured for the exosomes isolated with miRCURY (blue), ExoQuick (green), TEIR (red), and UC (yellow) from 5 ml, 1 ml, 500 μl, 250 μl, and 100 μl of human serum. The data in this graph are the mean values of the zeta potential ± SEM (n = 3)

**Fig 8:**
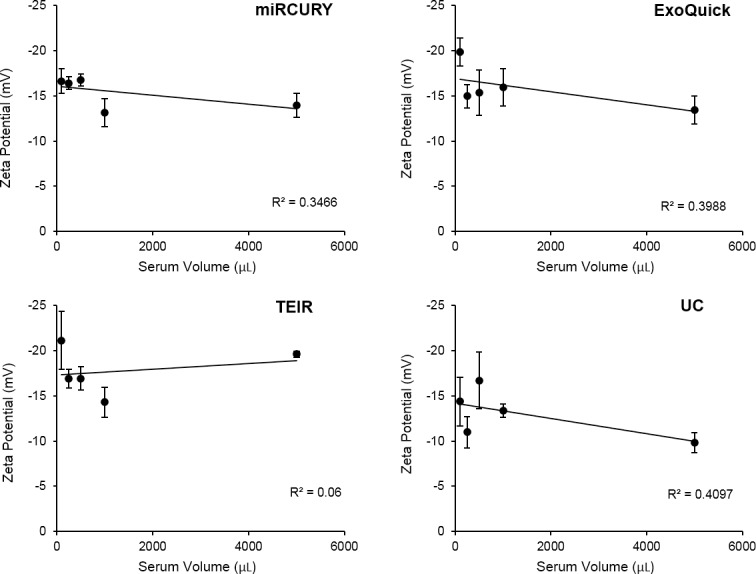
Correlation between the volume of serum and the zeta potential of the isolated solutions. The data in this graph are the mean values of three experimental replicates (n = 3) ± SEM. (A) miRCURY, (B) ExoQuick, (C) TEIR, and (D) UC.

Using the ZetaView instrument, the zeta potential was measured for exosome samples extracted with the four isolation techniques using the different serum starting volumes. Triplicates were isolated and measured for each volume and technique. All of the zeta potential measurements were negative and within the range of -9.80 to -21.1 mV at 23°C, as shown in **Fig 7**. We also examined the possiblecorrelation between the starting volume and the magnitude of the zeta potential for each technique, but no significant correlations were identified (**Fig 8**).

**Fig 9:**
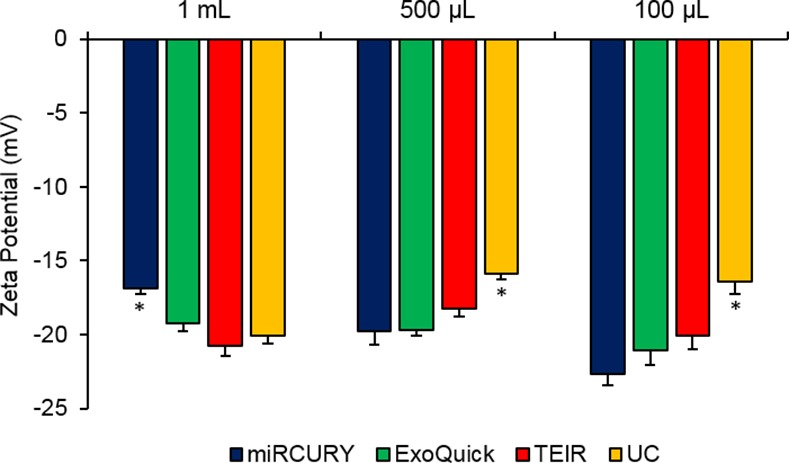
Zeta potential measurements for individual serum exosomes isolated from the different techniques and serum volumes. Using the ZetaView instrument, the zeta potential (mV) was measured for the exosomes isolated from individual human serum samples using miRCURY (blue), ExoQuick (green), TEIR (red), and UC (yellow) with starting volumes of 1 ml, 500 μl, and 100 μl. The data in this graph are the mean values of the zeta potential (n = 3) ± SEM, *p≤0.05.

To further confirm the zeta potential results with the pooled serum samples, we also measured the zeta potential of the exosomes isolated from the individual serum samples (**Fig 9**). The individual serum exosomes exhibited potentials similar to those from the pooled serum, having a range of -15.9 to -22.6 mV. Contrary to the pooled serum results, though, ANOVA tests revealed the zeta potentials of the individuals to be significantly different between the techniques for the 1 mL (p = 0.002), 500 μL (0.0002), and 100 μL (0.006). For the 1 mL, all techniques produced significantly higher magnitude potentials than miRCURY, with p-values of 0.008, 0.002, and 0.002 when comparing miRCURY to ExoQuick, TEIR, and UC. For the 500 μL and 100 μL isolates, miRCURY, ExoQuick, and TEIR isolated particles with significantly higher magnitude zeta potentials than UC, with post-hoc t-tests producing p-values of 0.01, 0.00003, and 0.006 for 500 μL and 0.0007, 0.009, and 0.02 for 100 μL, respectively.

### Exosomal RNA Isolation and Analysis

**Fig 10:**
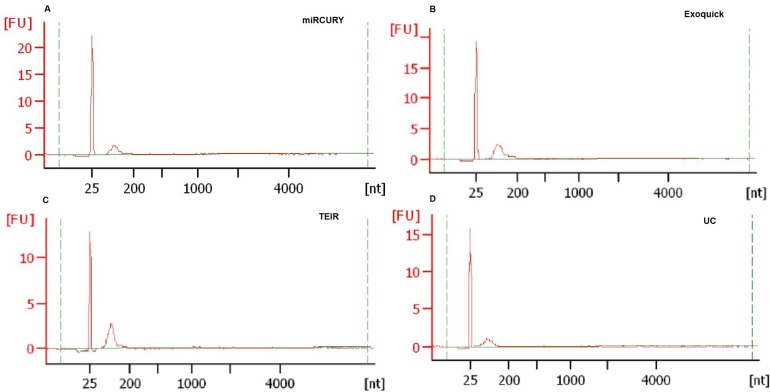
Characterization of exRNA extracted from exosomes isolated using the four techniques. exRNA quality was evaluated using the Agilent Bioanalyzer with RNA 6000 Pico kit for the exosomes extracted using the different isolation techniques and serum volumes. The y-axis represents fluorescence, and the x-axis is the size of the RNA, measured in nucleotides (nt). (A) miRCURY, (B) ExoQuick, (C) TEIR, and (D) UC.

**Fig 11:**
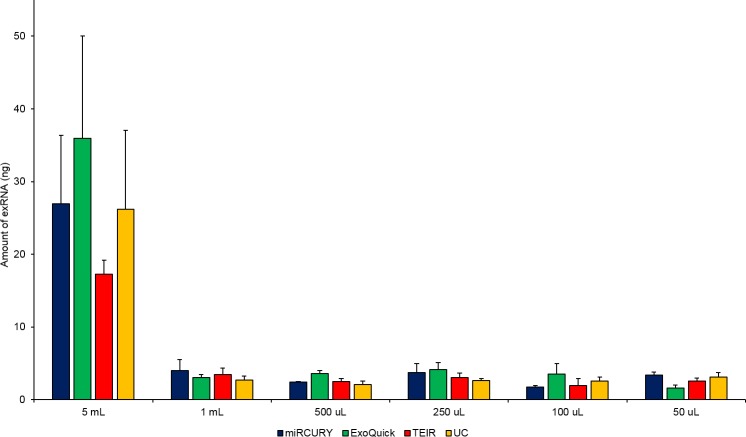
Quantity of exRNA extracted from exosomes isolated using the different techniques and serum volumes. exRNA concentration was measured using the Agilent Bioanalyzer with RNA 6000 Pico kit for the exosomes extracted using miRCURY (blue), ExoQuick (green), TEIR (red), and UC (yellow) with the six different serum volumes. Total amount of exRNA in each isolation was compared between different techniques. The data in this graph are the mean RNA amount (ng) of three experimental replicates (n = 3) ± SEM.

To compare the exRNA content of the exosomes between the different techniques, RNA was extracted from all exosome samples isolated using the various techniques and serum starting volumes. All extracted exRNA was shown to be of good quality, with a single RNA peak between 25 and 200 nucleotides and no signs of ribosomal 18S or 28S peaks, as illustrated in **Fig 10**. The concentration measurements were converted to exRNA yield for each sample, and these values are shown in **Fig 11**. No statistically significant differences were seen in the amount of exRNA isolated by the different techniques for each serum volume. While the exosomes isolated from 5 ml of pooled human serum contained greater than 3-fold more exRNA than those from the other serum volumes, there was no consistent trend between exRNA yield and starting volume.

**Fig 12:**
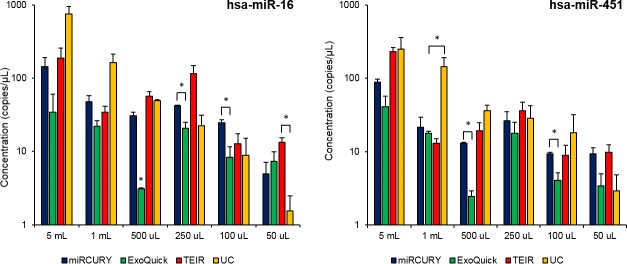
Expression of miR-16 and miR-451 in the exosomes isolated from the different techniques and volumes of serum. ddPCR was used to measure the absolute concentration of miR-16 and miR-451 in the exosomes isolated with miRCURY (blue), ExoQuick (green), TEIR (red), and UC (yellow) using the different volumes of human serum. The values in the graph are the mean concentration (copies/μl) ± SEM (n = 3), *p≤0.05.

After RNA isolation, we measured the concentrations of two miRNAs (hsa-miR-16 and hsa-miR-451) that are known to be present in serum exosomes and have been previously used to assess the efficiency of exosome isolation techniques [39]. We used ddPCR to evaluate whether these techniques significantly affect the expression profile of the isolated miRNA. With exosomes isolated from the different volumes of serum, we found that all techniques produced exosomes containing these miRNAs (**Fig 12**). A positive linear correlation was seen between serum volume and miRNA concentration for all techniques and both miRNAs. Comparing miR-16 concentration and serum volume, the R^2^ values were 0.9549, 0.6447, 0.6814, and 0.9982 for miRCURY, ExoQuick, TEIR, and UC, respectively. For the miR-451 linear correlations, the R^2^ values for miRCURY, ExoQuick, TEIR, and UC were 0.957, 0.8279, 0.9547, and 0.8692, respectively. The only significant difference in miRNA concentrations was within the measurements from 500 μL of serum for both miR-16 (p = 0.0007) and miR-451 (p = 0.001). For the miR-16 measurements, these significant differences were between miRCURY and UC (p = 0.03), and ExoQuick and miRCURY (p = 0.02), TEIR (p = 0.04), and UC (p = 0.002). With miR-451, only miRCURY and ExoQuick were statistically different (p = 0.0002).

## Discussion

In this study, we compared four different exosome isolation techniques (UC, ExoQuick, miRCURY, and TEIR) using six different starting volumes (5 ml, 1 ml, 500 μl, 250 μl, 100 μl, and 50 μl) of a pooled human serum, as well as three different volumes (1 ml, 500 μl and 100 μl) of six individual human donor samples. Using TEM imaging and NTA, we confirmed that all four technologies isolated particles within the size range of exosomes (40–150 nm) and with the traditionally reported morphology [43, 57, 58]. We further confirmed the identity of these particles as exosomes by analyzing the expression of exosome-enriched proteins, CD9 and CD63, through TEM immunogold staining and western blot. Our data showed that all samples expressed CD63 and CD9 similar to previous publications [58]. Quantitatively, we compared the exosomes isolated using the different techniques in terms of the physical properties of the particles, the particle yield, and the quality and quantity of their exRNA content. Overall, our study supports the feasibility of using these four methods to isolate large number of exosomes from different volumes of human serum samples. To our knowledge, this is the first study to integrate zeta potential and absolute miRNA quantification, using ddPCR as isolation technique comparison parameters.

With increasing knowledge of their role in cell-to-cell communication and their potential as biomarkers, the interest in the field of exosomes has grown dramatically over the past few years [19, 59, 60]. Before exosome research can be expanded any further, though, reliable and efficient techniques for isolating exosomes from limited heterogeneous biological samples, without disrupting the physical properties and contents of the particles, need to be identified. This report aims at comparing different techniques of exosome isolation using innovative modalities, including NTA using ZetaView and absolute miRNA quantification via ddPCR.

Using western blot and immunogold staining with TEM, we have confirmed the expression of two exosome proteins, CD63 and CD9. The protein expression data in this report contrasts the report by Zarvoni and colleagues [61], in which they reported lack of expression of exosome markers in the exosome samples isolated from serum by ExoQuick. The western blot in the aforementioned report showed intense, non-specific bands in the ExoQuick samples as compared to the samples isolated by UC. The authors explained this discrepancy by the presence of “spurious protein complexes” in the ExoQuick-derived samples. However, other reports have repeatedly shown protein expression from samples derived from ExoQuick and TEIR, as well as other PEG-related methods [58, 62]. Though levels of CD63 and CD9 expression from ExoQuick-derived samples varies among studies, none of them reported difficulty in the separation of the protein bands. We suggest that the non-specific protein expression in the report by Zarvoni et al. may be related to the protein samples preparation in terms of the type of lysis buffer used and the protein lysate preparation technique used, which may dramatically affect the degree of dissociation of the pelleted exosomes. Nonetheless, the authors did not state clearly in the methods section the means by which they prepared their western blot samples in terms of serum starting volume or the type of lysis buffer used. Lack of such crucial methodology information hampers proper comparison of data. However, protein expression levels alone cannot be used as the sole indicator of exosome yield [40, 46]. Our comprehensive analysis using protein expression data in combination with the size and morphology of the isolated particles adequately indicates that all techniques are able to isolate particles containing exosomes viable for down-stream research.

UC has been long adopted as the traditional technique for exosome isolation; however, this method is relatively tedious, time consuming, and technique sensitive, requiring prior training and the availability of an ultracentrifuge. Compared to the commercial kits, exosomes extracted by UC from the pooled human serum sample produces significantly larger particles, as indicated by the diameter of the particles. This fusion event is seen in **Fig 2**, with the size distribution curves of UC being shifted slightly to the right as compared to the commercial kits. As previously suggested, centrifuging at such high speeds possibly leads to fusion of the particles with contaminants and other proteins, affecting the physical properties of the exosomes and the sensitivity of proteomic analysis [10, 63, 64]. However, the phenomenon of isolating significantly larger diameter particles with UC was not observed in the individual serum samples. Nonetheless, the commercial kits showed consistency in the diameter and concentration of the extracted particles with both the single pooled serum sample and the individual serum samples, suggesting that these kits are a viable alternative for exosome isolation, even among variable human samples. Beside causing fusion of particles, applying different centrifugation forces over multiple cycles, though helpful in removing cell debris and other contaminants, can also cause loss of exosomes from the sample, leading to lower and more variable exosome yield [37]. This impact on exosome recovery in UC is congruent with the lower total number of particles seen in our UC samples compared to the kits, a trend which has also been previously observed by another group [38]. The more particles from the commercial reagents might be because PEG (polyethylene glycol)-based volume exclusion is used to precipitate non-exosome nanoparticles, such as serum protein aggregates, as supported by the lower exosome purity reported in a recent method-development study of ExtraPEG [41, 62].

We observed a linear correlation between the volume of the serum used for isolation and the total number of particles isolated. While the total number of particles isolated by ExoQuick in our study was comparable to that reported by Caradec et al., they showed that the yield of exosomes by UC remains constant regardless of the starting volume of serum [65]. This directly contrasts our results, with a 35-fold decrease in the total number of particles isolated from 5 ml to 50 μl of serum, though Caradec et al. used a much smaller starting sample volume range than we did in our study [65].

Although variations in zeta potential existed between different isolation techniques and starting sample volumes, our zeta potential measurements show that the extracted exosomes by the different techniques are negatively charged and within the range previously reported by other groups [32, 66]. The biological significance of the variations between techniques and starting volumes, though, remain to be determined. The small magnitude of the nanoparticles’ zeta potentials indicate their instability in solution, suggesting the necessity of using caution when handling and storing exosomes at temperatures othen than -80°C [67–70].

Different exRNAs identified in exosomes have been suggested to play roles in exocytosis, angiogenesis, and tumorigenesis, so determining the exRNA quantity and quality between different isolation techniques is necessary [71–73]. Surprisingly, even though the kits isolated a significantly greater total number of particles than UC, this did not appear to affect the amount of extracted exRNA. The commercial kits yielded similar amount of exRNA for the majority of the serum starting volumes with UC. The expression levels of miR-16 and miR-451, measured using ddPCR, indicate that each isolation technique may produce a slightly different profile of exosome RNA. Generally, the miRNA concentrations, varied greatly within and among the different techniques and serum volumes. This miRNA yield variability has been previously reported by other groups [51, 55]. This emphasizes the necessity of using a consistent exosome isolation technique across different samples within the same study. While R^2^ values support the existence of an overall linear correlation between miRNA concentration and serum starting volume, a lower serum starting volume did not guarentee a lower miRNA concentration in our data. This disproportionate relationship that we observed between the concentration of particles and the concentration of miRNAs is probably due to the heterogeneity of exRNA distributions across the exosome populations and the potential contamination of non-exosome particles including high-density lipoproteins and RNA binding proteins (argonautes 1 and 2) aggregates [54, 74–76].

Despite all the positive factors of our study, we are aware of some limitations in our design. First, this study does not intend to cover all available exosome isolation reagents from the different providers; nonetheless, we selected three of the most commonly used kits in the field [77]. Second, this comparison used only human serum samples, thus it is necessary to perform similar studies using other body fluids, such as urine, aqueous humor, cerebrospinal fluid, and amniotic fluid. Third, we acknowledge that though the biological activity of extracted exosomes is of prime importance in verifying its clinical relevance, examining this activity in our extracted particles was beyond the scope of this study. Such an analysis, though, may be further explored in future studies. Fourth, we cannot exclude the possibility of lipoprotein contamination of our isolated particles as previously shown by Deregibus et al. [66] and Sodar and colleagues [78]. In this aspect, we strongly agree with the recent publication by Deregibus et al. that this contamination though represent a limitation for almost all precipitation-based exosome isolation techniques, should not be an obstacle against using these techniques for biomarker purposes. The intent of our study as well as others is not to discriminate RNA associated with lipoproteins from those associated with vesicles, but to identify the population of small RNA that can be used as a reliable biomarker. However, to counteract this limitation, which is inevitable with most isolation techniques, we recommend using consistent and repeated exosome isolation techniques within a given group of investigated biological samples in order to guarantee valid comparability. On the other hand, if obtaining a highly pure exosome population on the expense of the yield of exosomes is acceptable, restoring to methods based on pull-down techniques such as Exocarp is recommended [79–81]. Nonetheless, this option is not always attainable especially with limited biological samples.

In summary, our comprehensive study has supported the use of the commercial kits miRCURY, ExoQuick, and TEIR as an adequate alternative to UC, even with limited amounts of heterogeneous biological starting material. Having multiple, less tedious alternatives to isolate exosomes will promote more exosome-related studies, helping better understanding of the functions of exosomes and possibly identifying new biomarkers and drug delivery systems. Our study will help researchers in selecting the best isolation technique for their purposes based on the amount of biological sample available and the necessary downstream analyses.
